# Older and Wiser: Interpretation of Proverbs in the Face of Age-Related Cortical Atrophy

**DOI:** 10.3389/fnagi.2022.919470

**Published:** 2022-07-04

**Authors:** Vanja Kljajevic

**Affiliations:** Department of Neuromedicine and Movement Science, Faculty of Medicine and Health Sciences, Norwegian University of Science and Technology (NTNU), Trondheim, Norway

**Keywords:** cognitive aging, brain atrophy, middle-temporal gyrus, comprehension, figurative meaning, cognitive sex differences

## Abstract

In the present study, we investigated whether interpretation of proverbs differs across the lifespan and if so, whether it is associated with age-related fronto-temporal atrophy. Using a sample of 333 healthy individuals aged 18–89 years, we found a significant effect of age on proverb interpretation [*H*(2) = 12.001, *p* = 0.002]: old adults (OA) were better than young adults (YA) (*p* = 0.002), and so were middle-aged-adults (MA) (*p* = 0.005). OA and MA had significantly less grey matter (GM) than YA in frontal and temporal lobes bilaterally, and OA less than MA in the right temporal lobe. GM volumes in these regions did not moderate the effect of age on the proverbs scores. The whole-brain analysis of groups’ GM maps revealed that the proverbs scores were associated with more GM in YA relative to OA in the right middle temporal gyrus, which is consistent with evidence on the role of this area in processing of unfamiliar proverbs. Overall, our data suggest that interpretation of proverbs is well preserved in late adulthood, despite considerable age-related cortical atrophy.

## Introduction

Current research on typical cognitive aging is often focused on age-related cognitive decline, although it is equally important to understand why some abilities remain intact or improve with age, despite considerable age-related brain atrophy (Salthouse, 2009; Shafto et al., 2020). A pattern of typical cognitive aging that has been replicated many times so far consists of well-preserved vocabulary and general knowledge across the lifespan, and nearly linear decline of fluid cognitive abilities starting in early adulthood (Salthouse, 2010). In fact, substantial evidence shows that vocabulary and general knowledge do not just resist decline; they improve over time even in old age (Meinz and Salthouse, 1998; Park et al., 2002).

One type of verbal knowledge that has not been studied to the same extent as general vocabulary, and even less so in the context of aging, are proverbs. These fixed expressions convey well-known truths, shared values, and wisdom of a society, representing an important component of cultural literacy (Gibbs and Beitel, 1995). Dictionaries of proverbs may contain thousands of entries^1^ and require frequent updating, to incorporate novel items and imports from other languages. Proverbs have a form of full sentence and figurative meanings, although such expressions could be true if interpreted literally (e.g., *Many leaks sink a ship*). Familiar proverbs are stored in memory presumably as single units, like lexical items, whereas interpretation of unfamiliar proverbs (e.g., *The friendliest cats have the sharpest claws*) requires construction of meaning that relies on higher cognitive abilities, such as problem solving (van Lancker, 1990; Temple and Honeck, 1992). Since familiar proverbs are fixed expressions, one would expect that they are well preserved in the course of aging.

However, there is currently no consensus on how we interpret proverbs. The standard pragmatic model postulates sequential processing: a proverb’s literal meaning is considered first and once it is ruled out as inconsistent with the context, a figurative meaning is constructed (Grice, 1975; Searle, 1979). Yet, behavioral indices of proverb interpretation, such as reading and response times, do not support this view. Instead, they suggest that cognitively healthy people construct a figurative meaning within first few words of a proverb, and that it does not take longer to derive figurative than literal meanings (Ferretti et al., 2020). The constraint-based models postulate that literal and figurative meanings are activated simultaneously; the competition between the meanings is resolved based on the features available in the context (syntactic, lexical, conceptual, or other information) (McRae et al., 1998). Considering that proverb interpretation depends on cognitive flexibility, response inhibition, and updating of representations in working memory (Uekermann et al., 2008), and that these executive functions are affected by aging (Oschwald et al., 2019), one would expect more difficulties in selecting the correct interpretation in older than in younger people, even in familiar proverbs.

Research on the effects of aging on interpretation of proverbs has resulted in mixed findings. A longitudinal study spanning approximately 3 years and including 16 cognitively healthy individuals (Mini Mental State Examination score ≥ 24) aged 80–95 years showed that interpretation of familiar proverbs improved with age (Ulatowska et al., 1998). In contrast, evidence from several cross-sectional studies suggests a negative effect of aging on proverb interpretation in cognitively healthy people (Nippold and Uhden, 1997; Uekermann et al., 2008; Szepietowska and Filipiak, 2021). For instance, a study with 353 individuals aged 13–79 years reported that interpretation of familiar proverbs “reached a plateau” in the 20s, it was stable in the 30s, 40s, and 50s, but it began to decline in the 60s, with a significant decline being detectable in the 70s (Nippold and Uhden, 1997). The findings suggesting that proverb interpretation is worse in elderly than in younger individuals stand in stark contrast to the idea that verbal knowledge is preserved well into late adulthood (Salthouse, 2010).

Another important aspect of proverb interpretation across the lifespan pertains to age-related brain atrophy. Considering that interpretation of proverbs involves various cognitive processes, it is expected to engage multiple brain areas. Lesion and functional neuroimaging studies have suggested involvement of a range of brain areas and white matter tracts in proverb interpretation, from fronto-temporal regions (Yi et al., 2017) to corpus callosum (Rehmel et al., 2016) and the cerebellum (Bohrn et al., 2012). Still, the question of how reduction of GM volumes in typically aging brains (Oschwald et al., 2019) relates to interpretation of proverbs remains unclear.

Considering the discrepancies in findings on proverb interpretation in the course of aging, on the one side, and lack of understanding of the relationship between proverb interpretation and reduction in GM volumes in the aging brain, on the other, the present study investigated whether interpretation of these expressions would differ in cognitively healthy older individuals in comparison to middle-aged and young people, and whether age-related cortical atrophy would be associated with proverbs scores. To establish cortical atrophy in older participants, we tested for group differences in GM volumes of the left and right frontal and temporal lobes. We deliberately chose lobar volume measures of GM because they are sufficiently informative as an indicator of age-related atrophy of the relevant regions, even though they may appear crude compared to selecting specific subregions (Gogtay and Thompson, 2010). We included these regions of interest (ROIs) bilaterally, because (a) both hemispheres support figurative language processing (Uekermann et al., 2008), (b) language processing in general is more bilateral in women than in men (Kimura, 1983; Kljajevic, 2022), and (c) lateralization reduces with age, probably due to compensatory recruitment (Cabeza et al., 2002).

Assuming the prototypical cognitive aging profile (Salthouse, 2010), we expected older adults to have preserved interpretation of proverbs. Based on previous findings suggesting more pronounced atrophy with advanced age (Oschwald et al., 2019), we expected to find significant reduction in GM volumes in older compared to young and middle-aged participants.

## Materials and Methods

### Data

Data for the present study were obtained from the Cambridge Center for Aging and Neuroscience (CamCAN) repository http://www.mrc-cbu.cam.ac.uk/datasets/camcan/ (Shafto et al., 2014; Taylor et al., 2016). The study followed the Helsinki Declaration guidelines for studies with human subjects and was approved by the local ethics committee (Shafto et al., 2014; Taylor et al., 2016).

### Participants

The sample (*N* = 333) consisted of 41 young adults (YA) aged 18–30 years, 139 middle-aged (MA), 31–60 years, and 153 older adults (OA), 61–89 years of age. All participants had available scores on the proverb test (Table 1) and all but seven had available neuroimaging data.

**TABLE 1:** Participants’ characteristics and scores on the proverb test.

	YA (*n* = 41)	MA (*n* = 139)	OA (*n* = 153)
Age range	18–30	31–60	61–89
Age (mean, SD)	24.99 (± 3.65)	47.04 (± 8.35)	72.41 (± 7.52)
Gender (F/M)	23/18	65/74	74/79
Handedness	77.73 (± 46.63)	75.84 (± 54.1)	78.27 (± 52.9)
Score	3.98 (± 1.69)	4.83 (± 1.5)	4.83 (± 1.6)

### Behavioral Task

The task was to read and interpret three common English proverbs shown consecutively on the screen using E-Prime. Participants’ responses were recorded for up to 60 s for each trial. After each trial, the experimenter provided a score for that trial. Incorrect or “don’t know” responses were scored zero, responses that interpreted the proverbs literally (i.e., concrete meaning) were considered partly correct and scored 1, and responses that interpreted the proverbs’ meanings as abstract were scored 2. The total score was calculated by adding up scores on all trials, with a possible score range of 0–6.

### MRI Data: Acquisition and Preprocessing

For details on the CamCAN neuroimaging protocol see Taylor et al. (2016). Briefly, data were collected at a single site using a 3T Siemens TIM Trio scanner 32-channel head coil. High resolution structural T1-weighted images were obtained using a Magnetization Prepared RApid Gradient Echo (MPRAGE) sequence, with the following parameters: Repetition Time (TR) = 2,250 ms, Echo Time (TE) = 2.99 ms, flip angle = 9 degrees, field of view (FOV) = 256 × 240 × 192 mm, voxel size 1 × 1 × 1 mm, GRAPPA acceleration factor = 2, acquisition time = 4 min and 32 s.

The MRI data preprocessing was performed using Statistical Parametric Mapping (SPM8, Wellcome Trust Center for Neuroimaging) implemented in MATLAB R2007a (MathWorks, Natick, MA). T1-weighted images were first realigned so that the origin of each T1 scan was set to the AC/PC line. Using New Segment, images were then segmented into six tissue classes (gray matter, white matter, cerebrospinal fluid partitions, skull, soft tissue outside the brain, and air/other stuff outside the head). DARTEL was used to obtain non-linear deformations for warping grey matter (GM) and white matter (WM) images (Ashburner, 2007). The DARTEL-imported versions of GM and WM maps were used to generate flow fields and a series of average templates to which the data were iteratively aligned. The final template, which was registered to MNI space by an affine transformation, and the flow fields obtained in the previous step, were applied to the native GM maps. The spatially normalized GM maps were smoothed with a 10 mm full-width-at-half-maximum (FWHM) Gaussian kernel.

ROIs were determined using WFU Pick atlas: left and right frontal and temporal lobes. Their values were extracted from the warped GM maps using an in-house algorithm and normalized by total intracranial volume (TIV), as an index of head size (Barnes et al., 2010). TIV was calculated by summing up GM, white matter (WM), and cerebrospinal fluid (CSF).

### Statistical Analyses

Kruskal-Wallis test was used to compare the groups in age, handedness scores, ROI values, TIV, as well as proverbs scores, which was followed up by pairwise comparisons to establish which groups differed. The effect sizes (*r*) for the pairwise tests were calculated by dividing the *z* scores with the squared root of N (Field, 2018). Pearson χ^2^-test was used to test for possible differences in sex distribution among the groups. Associations between participants’ age, proverbs scores, and ROIs values were assessed by using Spearman test, and possible sex differences in proverbs scores and ROIs values within each group by Mann-Whitney test. An alpha level was set at 0.05 for all statistical tests, unless otherwise stated. All tests were two-tailed. These analyses were performed in Statistical Package for Social Science 22 (SPSS) (IBM SPSS Statistics for Windows, Version 22.0, Armonk, NY, United States, IBM Corp. Released 2013). To determine whether the effect of age on proverbs score was moderated by ROIs GM values, we performed a set of moderation analyses in PROCESS 3.5.3 www.afhayes.com (Hayes, 2018), implemented in SPSS. Parameters were set to determine bias-corrected bootstrap confidence intervals based on 5,000 bootstrap samples and the confidence interval was set at 95% value. Standard errors were corrected for heteroscedasticity using HC3 approach (Hayes and Cai, 2007). All variables were mean-centered.

Voxel-based analysis of GM maps between pairs of groups, with proverbs scores as a covariate of interest, and sex and TIV as covariates of no interest, were performed using full-factorial model in SPM8. The results were assessed at a statistical threshold of 0.05, corrected for multiple comparisons by using familywise error (FWE) rate, at a cluster size of k ≥ 20 contiguous voxels.

## Results

The groups differed significantly in age [*H*(2) = 273.553], with the adjusted *p*s for all pairwise comparisons being < 0.001. There were no statistically significant differences in handedness scores or in sex distribution among the groups.

**FIGURE 1:**
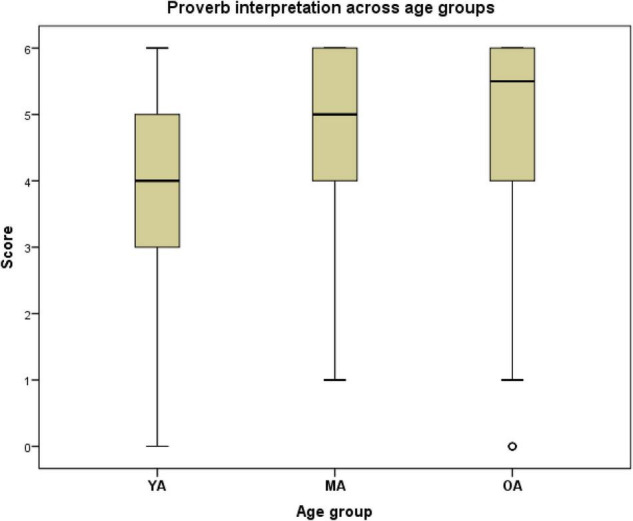
Proverb interpretation. YA, young adults; MA, middle-aged adults; OA, old adults.

There was a significant effect of age on participants’ interpretation of proverbs [*H*(2) = 12.001, *p* = 0.002]. As the boxplot of the data shows, the medians were higher in both OA and MA than in the YA group, and higher in the OA group than in the MA group (Figure 1). Follow-up pairwise comparisons showed that these differences were statistically significant after adjusting the *p*-values for multiple comparisons (YA vs. MA: *p* = 0.005, *r* = –0.23; YA vs. OA: *p* = 0.002, *r* = –0.24). The difference between MA and OA’s proverbs scores was not statistically significant. There were no statistically significant sex differences in proverb interpretation, ROI volumes, or TIV within any group.

As expected, there were statistically significant group differences in GM volumes of frontal and temporal lobes, which survived a correction for multiple comparisons (Table 2). Specifically, OA and MA had significantly less GM volume in each ROI than YA, and OA had significantly less GM volume in the right temporal lobe than MA. The observed effects were small to medium. The groups did not differ significantly in TIV [*H*(2) = 1.553, *p* = 0.460, n.s.].

**TABLE 2:** Group differences in GM volumes in ROIs.

ROI	YA vs. OA	YA vs. MA	MA vs. OA
Left frontal *H(2)* = 27.633, *p* < 0.001	*p* < 0.001, *r* = 0.375	*p* = 0.001, *r* = 0.275	*p* = 0.084, *r* = 0.128
Right frontal *H(2)* = 26.249, *p* < 0.001	*p* < 0.001, *r* = 0.367	*p* = 0.001, *r* = 0.280	*p* = 0.162, *r* = 0.113
Left temporal *H(2)* = 17.137, *p* < 0.001	*p* < 0.001, *r* = 0.289	*p* = 0.038, *r* = 0.221	*p* = 0.083, *r* = 0.129
Right temporal *H(2)* = 33.071, *p* < 0.001	*p* < 0.001, *r* = 0.386	*p* = 0.013, *r* = 0.213	*p* = 0.001, *r* = 0.213

*The p-values have been adjusted for multiple comparisons.*

Significant negative associations between age and ROI values survived Bonferroni correction for multiple comparisons (left frontal: *r*_*s*_ = –0.263, right frontal: *r*_*s*_ = –0.248, left temporal: *r*_*s*_ = –0.196, right temporal: *r*_*s*_ = –0.267, with all *p*s < 0.001). A series of moderation analyses with age as focal predictor, proverbs scores as the outcome variable, and each ROI as a moderator showed that ROI volumes did not significantly affect the impact of age on the scores.

**FIGURE 2:**
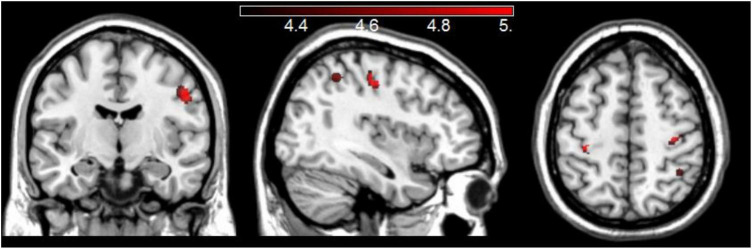
Areas with more GM in YA compared to OA when considering the groups’ proverbs scores and adjusting for sex and TIV.

The whole-brain analysis of groups’ GM maps with proverbs scores as covariate of interest, and sex and TIV as covariates of no interest, revealed clusters with significantly more GM volume in YA than in OA in the right middle-frontal gyrus and in the precentral and postcentral gyri bilaterally. These clusters survived a correction for multiple comparisons at FWE *p* < 0.05 (Figure 2).

## Discussion

The main finding of the present study suggests that interpretation of proverbs improves with age, as OA interpreted proverbs better than MA and YA, and MA better than YA. This finding is consistent with the prototypical cognitive profile (Salthouse, 2010), in which general knowledge and vocabulary improve with advancing age. It is also consistent with findings from Ulatowska et al.’s (1998) longitudinal study, but not with findings from the cross-sectional studies that suggest that this ability declines with age (Nippold and Uhden, 1997; Szepietowska and Filipiak, 2021). The discrepancies in findings in these studies could be due to several reasons, one of which might be the difference in task demands. For example, in Nippold and Uhden’s (1997) study each proverb appeared at the end of fourth sentence in a short story and participants were required to interpret the proverb after hearing the story. Despite the shortness and simplicity of the stories, this task is more complex and likely more taxing on cognitive resources than the task in which participants are required only to retrieve meanings of familiar proverbs from the long-term memory, as in the present study. In Szepietowska and Filipiak’s (2021) study, the 70+ years old group had a MoCA score of 23 (± 1.3), which is below the cut-off score that indicates healthy cognitive status (≥ 26), and therefore there is a possibility that factors other than advanced age might have contributed to the results, such as onset of dementia (Kljajevic, 2015; Boyle et al., 2021).

Since there is a great deal of variability in determining age groups in the literature on aging, to test whether the pattern we observed when including 60+ years old people in the OA group would also emerge in an older OA group, we conducted the same analysis including only participants 65+ years of age in the OA group. This analysis showed a significant effect of age on proverbs scores [*H*(3) = 8.548, *p* = 0.036], and higher proverb scores in the OA 65+ group than in the YA group, which remained statistically significant after adjusting for multiple comparisons (*p* = 0.033).

Poorer interpretation of proverbs in young relative to middle-aged and older people in the present study is consistent with another finding suggesting lower cultural literacy in younger participants, which pertains to knowledge of names of famous people (Kljajevic and Erramuzpe, 2018, 2019). Furthermore, a study involving American college students showed that they interpreted proverbs more freely, disregarding the fixed meanings of these expressions listed in dictionaries of proverbs. For instance, they interpreted the proverb *The rolling stone gathers no moss* in three ways: (1) As an analogy with a constantly “on” machine; (2) in a positive sense, i.e., moving was seen as a sign of freedom and (3) in a negative sense, as something that prevents a person to settle down (Gibbs and Beitel, 1995). Thus, although perhaps surprising, our finding that younger people interpreted proverbs less accurately than older participants is well aligned with the disregard of proverbs’ fixed meanings found in the study with American college students.

Consistent with previous findings on age-related brain atrophy, older participants in our study had significantly reduced GM volumes relative to YA. However, an unexpected finding based on comparisons of voxel-based morphometry measurements between OA and YA links more GM volume in the right middle-temporal gyrus, and pre- and post-central gyri bilaterally, with lower proverbs scores. The direction of this relationship is surprising, because greater GM volumes in frontal and temporal areas are typically associated with better performance on tests of crystalized abilities, such as retrieval of information from stored knowledge (Hoffman et al., 2017). Nevertheless, some studies reported the opposite pattern. For instance, de Zubicaray et al. (2011) found that better semantic ability in cognitively healthy older adults was associated with reduced GM volumes in a predominantly left-hemisphere network, which included the middle temporal gyrus. A recent study comparing brain activation patterns during interpretation of familiar and unfamiliar proverbs reported that unfamiliar proverbs activated the middle-temporal gyrus bilaterally, among other areas (Bohrn et al., 2012). The authors explained the activation of this area in interpretation of unfamiliar proverbs in terms of neural support in semantic integration and sentence processing, which is consistent with the finding on association of this area with lower accuracy in the interpretation of proverbs in YA in our sample.

The strengths of the present study are a relatively large sample, a similar number of men and women within each age group, and simplicity of the task. The limitations of the study are not accounting for possible effects of education, socio-economic status, and other factors that contribute to cognitive reserve (Stern, 2009), as well as a small number of proverbs. Although psychologists, psychiatrists, and neuropsychologists often use small number of proverbs for evaluative and diagnostic purposes (e.g., Delis et al., 1984; Torralva et al., 2009; Roca et al., 2010; Arcara and Bambini, 2016, but see Gorham, 1956, for a different approach), one drawback is that such tests underutilize internal features of proverbs. Owing to their different internal features, different types of proverbs may tap into different cognitive processes. For example, metaphorical proverbs (e.g., *Fair play is a jewel*), synecdochic (*The further the sight, the nearer the rain*), metonymic (*Fear gives wings*), hyperbolic (*All is fair in love and war*), and paradoxical proverbs (*Fair is not fair, but that which pleases*) may present different degrees of difficulty in interpretation. In typical cognitive aging, processing different types of proverbs may reveal differences not only in accuracy scores, but also in response times and in neural activation patterns.

Perhaps more importantly, different types of patients may tend to concretize meanings of some, but not other types of proverbs, which may distort the picture of their overall ability to interpret figurative language and thus compromise the evaluation. Psychologists, psychiatrist, and neuropsychologists since the early twentieth century have used proverbs to assess categorization and reasoning in various patient populations as well as in normal development (Harrow et al., 1974; van Lancker, 1990; Chapman et al., 1997; Thoma and Daum, 2006; Kaiser et al., 2013; Murphy et al., 2013). Impaired comprehension of proverbs has been confirmed in schizophrenia (Gorham, 1956; Kiang et al., 2007; Bambini et al., 2020), mild cognitive impairment (Leyhe et al., 2011; Cardoso et al., 2014), Alzheimer’s disease (Chapman et al., 1997), other types of dementia (Kaiser et al., 2013), chronic frontal lesions (Roca et al., 2010; Murphy et al., 2013), and aphasia (Chapman et al., 1997). Different types of interpretative errors in proverb comprehension are consistent with different conditions, as they may reveal thought disorder (e.g., in schizophrenia), tendency to concretize (e.g., in dementia, frontal lobe lesions), or more fundamental language dysfunction (as in post-stroke aphasia). New insights of differential error patterns in proverb interpretation across patient populations would enhance current understanding of proverbs processing.

## Conclusion

In conclusion, the present study supports the notion that interpretation of proverbs improves during the course of healthy aging, despite significant fronto-temporal age-related atrophy. Our findings also suggest no sex differences in this type of knowledge, regardless of age. Longitudinal studies with large samples and access to data relevant to cognitive reserve, such as education, socio-economic status, and IQ, need to be conducted to determine whether interpretation of different types of proverbs leads to different comprehension patterns across the lifespan.

## Data Availability Statement

Publicly available datasets were analyzed in this study. This data can be found here: https://camcan-archive.mrc-cbu.cam.ac.uk/dataaccess/.

## Ethics Statement

The CamCAN study followed the Helsinki Declaration guidelines for studies involving human subjects and was approved by the local Ethics Committee.

## Author Contributions

The author confirms being the sole contributor of this work and has approved it for publication.

## Conflict of Interest

The author declares that the research was conducted in the absence of any commercial or financial relationships that could be construed as a potential conflict of interest.

## Publisher’s Note

All claims expressed in this article are solely those of the authors and do not necessarily represent those of their affiliated organizations, or those of the publisher, the editors and the reviewers. Any product that may be evaluated in this article, or claim that may be made by its manufacturer, is not guaranteed or endorsed by the publisher.

## Footnotes

1 For instance, the 6th edition of The Oxford Dictionary of Proverbs contains 2,500 entries (Speake, 2015).
